# Cartilage thickness at the apex of femoral resections in kinematically aligned total knee arthroplasty is close to 2.5 millimeters

**DOI:** 10.1002/jeo2.70641

**Published:** 2026-02-19

**Authors:** Saahil Sandhar, Alexander J. Nedopil, Maury L. Hull

**Affiliations:** ^1^ School of Medicine University of California Davis Sacramento CA USA; ^2^ Department of Orthopaedic Surgery Konig‐Ludwig‐Haus, University of Wurzburg Wurzburg Germany; ^3^ Institute for Orthopaedic Surgery and Research, College of Medicine California Northstate University Elk Grove CA USA; ^4^ Department of Biomedical Engineering University of California Davis Davis CA USA; ^5^ Department of Mechanical Engineering University of California Davis Davis CA USA; ^6^ Department of Orthopaedic Surgery University of California Davis Medical Center Sacramento CA USA

**Keywords:** kinematic alignment, knee replacement, magnetic resonance, osteoarthritis initiative

## Abstract

**Purpose:**

Determining the correct resection thickness on worn femoral surfaces in kinematically aligned (KA) total knee arthroplasty (TKA) is uncertain since cartilage thickness is unknown. In the past, a 2 mm fixed adjustment has been used, but this may not reflect cartilage thickness at the apices of resections. Our objectives were to determine apex thickness for each resection based on a new method involving MR images, compare this thickness to direct measurements using calibrated photographs, and determine whether an increase in the 2 mm fixed adjustment is a refinement of interest to an already highly successful surgical procedure.

**Methods:**

MR images from 100 knees without evidence of OA were accessed in the Osteoarthritis Initiative (OAI) database. After aligning images in kinematic planes, the apices of the distal and posterior femoral resections were identified, and the apex cartilage thickness was computed.

**Results:**

From MR images and calibrated photographs, respectively, apex cartilage thickness was 2.3 mm ± 0.5 mm vs. 2.6 mm ± 0.7 mm for the distal medial resection, 2.3 mm ± 0.4 mm vs. 2.7 mm ± 0.6 mm for the distal lateral resection, 2.4 mm ± 0.5 mm vs. 2.7 mm ± 0.6 mm for the posterior medial resection, and 2.4 mm ± 0.6 mm vs 2.5 mm ± 0.5 mm for the posterior lateral resection. Mean differences were statistically significant (*p* ≤ 0.0035) except for the posterior lateral cartilage thickness (*p* = 0.1498).

**Conclusions:**

Apex cartilage thickness for MR image measurements was closer to 2.5 mm than 2 mm for all four resections. Apex cartilage thickness for calibrated photographs was greater than 2.5 mm and was comparable for all four resections. Although unlikely to affect patient‐reported outcome measures, a fixed adjustment for worn cartilage on the femur in KA TKA of 2.5 mm is a refinement of interest to bring more patients closer to the ideal alignment.

**Level of Evidence:**

N/A.

## Graphical abstract

Histograms showing cartilage thickness at the apices of four femoral resections based on analysis of MR image. Mean thickness was closer to 2.5 mm than 2 mm in all resections.

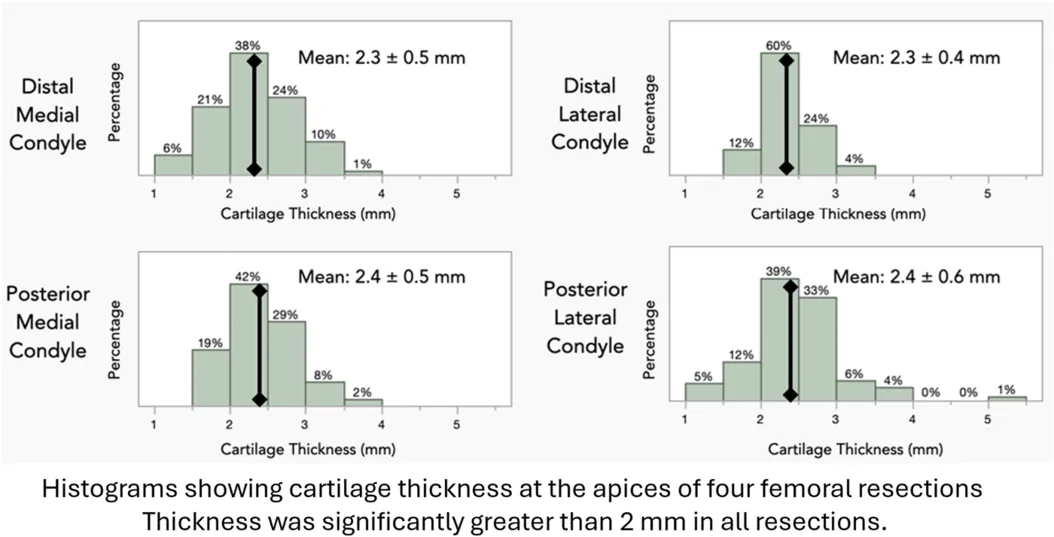

**Abbreviations**
ANOVA: analysis of variance
cLF: central region of the lateral femur
cMF: central region of the medial femur
DESS: double‐echo steady‐state
ICC: intraclass correlation coefficients
KA: kinematic alignment
MPR: multiplanar reformation
MR: magnetic resonance
MRI: magnetic resonance images
OA: osteoarthritis
OAI: osteoarthritis initiative
PCL: posterior cruciate ligament
PROMs: patient‐reported outcome measures
TKA: total knee arthroplasty

## INTRODUCTION

**Figure 1:**
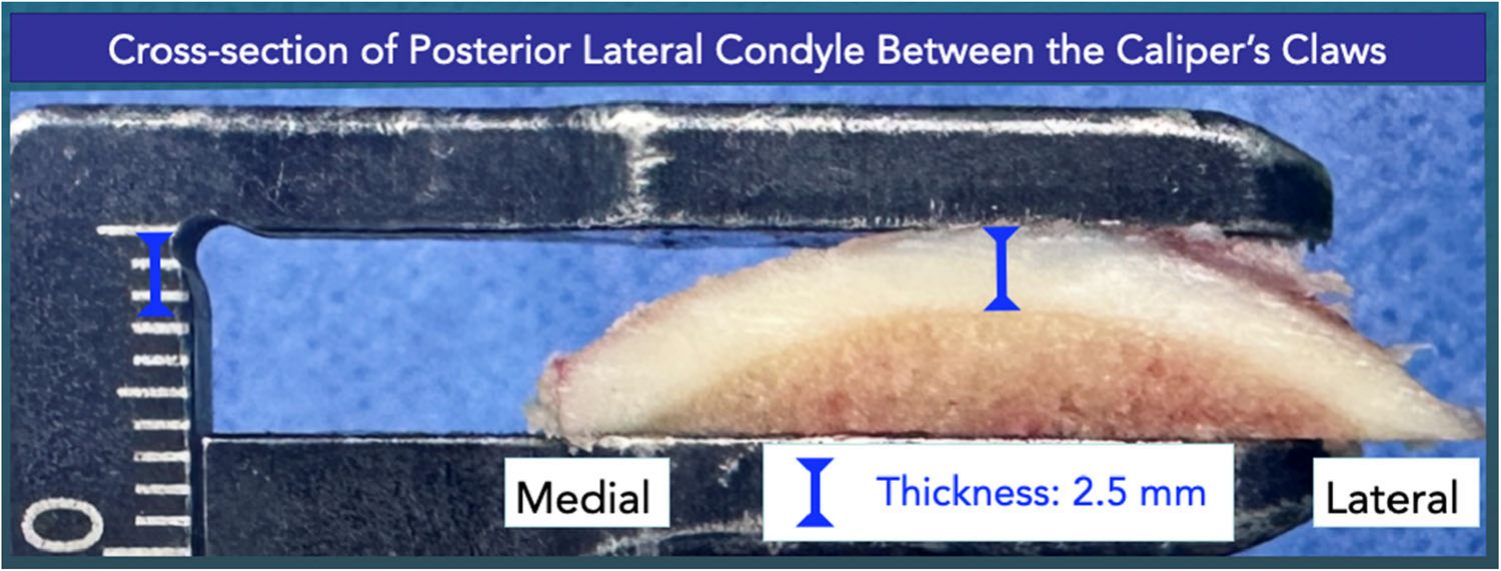
Image of femoral resection bisected in the medial‐lateral direction showing the convexity of the articular surface. Cartilage thickness must be known at the apex and perpendicular to the resection plane.

When performing kinematically aligned (KA) total knee arthroplasty (TKA), a goal is to restore native limb and knee alignments regardless of degree of preopertiave deformity and/or flexion contracture. Restoring native femoral alignments requires that the thickness of each femoral resection matches the corresponding thickness of the femoral component after adjustment for saw blade kerf and articular cartilage wear if present. To adjust for wear, the unworn (i.e. prearthritic) cartilage thickness is needed. Since resections are convex and since the femoral resection thickness is measured at its apex (Figure 1), unworn cartilage thickness must be known at this location. Previously, a fixed cartilage thickness of 2 mm has been used since this is the mean thickness from MR images [13]. Since this thickness may not have been measured at the apices of femoral resections however, cartilage thickness should be measured at these locations to determine the adjustment for femoral cartilage wear.

Using MR images to obtain these measurements rather than other methods offers several advantages. One is that large sample sizes are enabled by accessing the Osteoarthritis Initiative (OAI) database [18]. By concentrating on MR images for Kellgren‐Lawrence Grade 0, another is that cartilage thickness can be determined at the apices of all four femoral resections without the possible confounding effects of OA affecting cartilage thickness [2, 17]. Notwithstanding these advantages, measuring cartilage thickness from MR images has a number of limitations which call the ability to obtain accurate thickness measurements into question [3].

Several previous studies have measured cartilage thickness during surgery at the apices of femoral resections [3, 8, 14]. However, no previous study measured thickness at the apices of femoral resections based on MR images and no previous study reported values for each resection. Thus, it is unknown whether measurements of cartilage thickness at the apices of resections from MR images yield reliable information and whether the same adjustment for worn cartilage can be used for each resection.

Our objectives were to use a new method based on MR images to determine the apex cartilage thickness for each resection, to compare the apex thickness based on MR images to direct measurements reported previously using calibrated photographs [14], and to determine whether an increase in the 2 mm fixed adjustment is a refinement of interest to an already hightly successful surgical procedure. The comparison to direct measurements from calibrated photographs addressed the reliability of thickness measurements at apices of femoral resections from MR images. Our hypothesis was that apex cartilage thickness based on MR images would be significantly greater than 2 mm.

## METHODS

MR images of 100 patients were selected from the OAI database. Only patients without OA were included (i.e. Kellgren‐Lawrence grade of 0). Only patients with double‐echo steady‐state (DESS) images obtained from a 3 T MRI machine from Siemens (repetition time/echo time, 16.2/4.7; field of view, 14 cm; matrix, 307 ×348; bandwidth, 62.5 kHz) were used.

**Figure 2:**
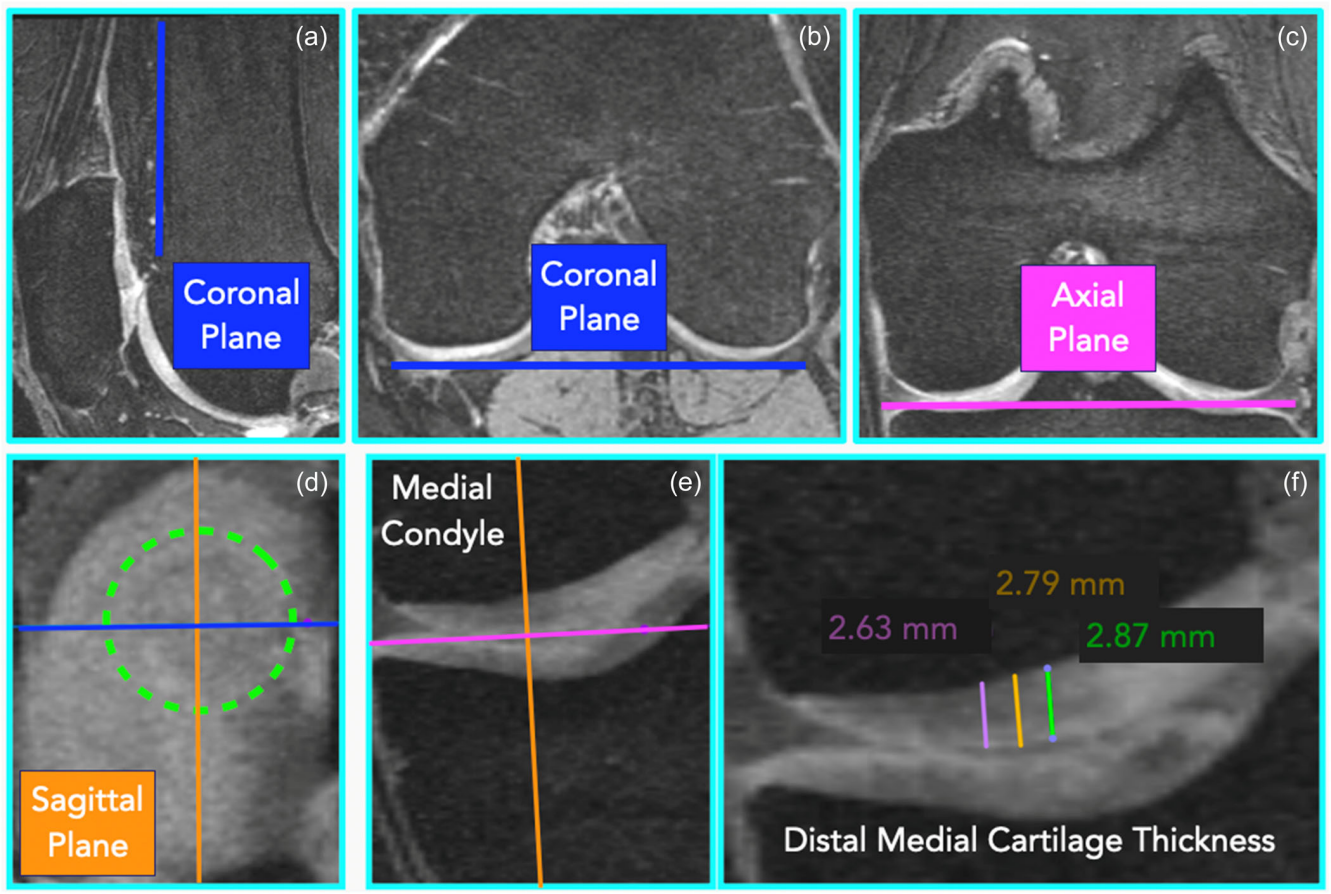
Composite of MR images showing the method of measuring cartilage thickness at the apex of a femoral resection. (a) Setting sagittal orientation of coronal plane (blue line) parallel to anterior femoral cortex. (b) Setting axial orientation of coronal plane parallel to posterior condyles. (c) Setting coronal orientation of axial plane (magenta line) parallel to distal condyles. (d) Localising the most distal slice, which visualises the distal medial cartilage (green circle), with the crosshair of the sagittal (orange line) and coronal planes positioned in the centre of the distal medial cartilage. (e) In the coronal view, showing the axial plane tangent to the most distal region of the distal medial cartilage, and the sagittal plane perpendicular to the resection plane. (f) Measuring cartilage thickness of the distal medial condyle parallel to the sagittal plane at three locations, which are 1 mm apart.

To measure the distal and posterior femoral cartilage thickness according to the resections performed with KA TKA, the following three steps were necessary. First, the imaging planes were oriented according to the kinematic planes (Figure 2a–c). Second, the most distal and most posterior points (i.e. apices) of the distal and posterior femoral resections, respectively, were found (Figure 2d–e). Third, cartilage thickness was measured at nine locations in a matrix centred on the most distal and posterior points (Figure 2f). Each of these steps is described in turn below.

To orient the imaging planes according to the kinematic planes, MR images were opened in the multiplanar reformation (MPR) window in the HOROS Dicom Viewer (https://horosproject.org) which displays the MR images in the sagittal, axial and coronal views. First, in the sagittal view, the kinematic coronal plane was set parallel to the anterior femoral cortex (Figure 2a), and in the axial view parallel to the posterior condyles (Figure 2b). To verify that the kinematic coronal plane was parallel to the posterior condyles, images were scrolled in the coronal view anteriorly and posteriorly to ensure that the posterior condyles appeared and disappeared in the same slice. Next, in the coronal view, the kinematic axial plane was set parallel to the distal femoral condyles (Figure 2c). To verify that the kinematic axial plane was parallel to the distal condyles, images were scrolled in the axial view proximally and distally to ensure that the distal condyles appeared and disappeared in the same slice. The kinematic axial and coronal planes are parallel to the planes of the distal and posterior resections, respectively. The kinematic sagittal plane was mutually perpendicular to the kinematic coronal and axial planes.

In the second step, to identify the most distal point of the distal medial condyle, images were scrolled proximally and distally in the axial view to identify the slice in which the cartilage of the distal medial condyle appeared and disappeared. In the most distal slice, which showed the distal medial cartilage, the crosshair of the sagittal and coronal planes was positioned in the centre of the visible cartilage (Figure 2d). Now, in the coronal view, the kinematic axial plane was tangent to the most distal point of the distal medial cartilage, and the kinematic sagittal plane was perpendicular to the resection plane (Figure 2e). The same steps were repeated for the distal lateral condyle.

To identify the most posterior point of the posterior medial condyle, images were scrolled anteriorly and posteriorly in the coronal view to identify the slice in which the cartilage of the posterior medial condyle appeared and disappeared. In the most posterior slice, which shows the posterior medial cartilage, the crosshair of the kinematic sagittal and axial planes was positioned in the centre of the visible cartilage. Now, in the axial view, the kinematic coronal plane was tangent to the most posterior point of the posterior medial condyle, and the kinematic sagittal plane was perpendicular to the resection plane. The same steps were repeated for the posterior lateral condyle.

In the third step, to measure the cartilage thickness at the apex of each distal resection, the measuring tool was used in the coronal view. A line was drawn parallel to the kinematic sagittal plane from where the kinematic axial plane contacted the cartilage to the subchondral bone (1^st^ measurement) (Figure 2f). Two parallel lines were drawn 1 mm medial and 1 mm lateral from the first line, extending from the distal cartilage to the subchondral bone (second and third measurements). The same steps were repeated in the coronal view, one slice more anterior and posterior (fourth to nineth measurements).

To measure the cartilage thickness at the apex of each posterior condyle, the measuring tool was used in the axial view. A line was drawn parallel to the kinematic sagittal plane from where the kinematic coronal plane contacted the cartilage to the subchondral bone (first measurement). Two parallel lines were drawn 1 mm medial and lateral from the first line, extending from the posterior cartilage to the subchondral bone (second and third measurements). The same steps were repeated in the axial view, one slice more proximal and distal (fourth to nineth measurements).

### Statistical analysis

Mean cartilage thickness at the apex of each femoral resection (henceforth termed mean apex thickness) was determined by computing the mean value for the nine measurements in the grid. The overall mean apex thickness was computed for each set of 100 resections.

A power analysis was performed based on a two‐sample *t*‐test with a level of significance of 0.05, a power of 0.95, a difference to detect of 0.5 mm, and a standard deviation of 0.5 mm (i.e. effect size = 1). The corresponding total sample size was 54. Unpaired *t*‐tests determined whether the overall mean apex thickness from the MR images differed significantly from those determined from the calibrated photographs. Also, 95% confidence intervals were computed for each overall mean apex thickness using each measurement method.

To assess repeatability and reproducibility, a two‐factor ANOVA was performed in which observer at three levels and subject at five levels were modelled as random effects. For each observer and subject, three trials were performed for each resection. The variance components for the observer, subject and error were used to determine intraclass correlation coefficients (ICC) for repeatability and reproducibility [1]. Repeatability was quantified as the standard deviation of the error variance. The standard deviation of the variance component for the observer quantified variability introduced by the observer. ICC values > 0.90 indicate excellent agreement and 0.75–0.90 indicate good agreement [11].

## RESULTS

**Figure 3:**
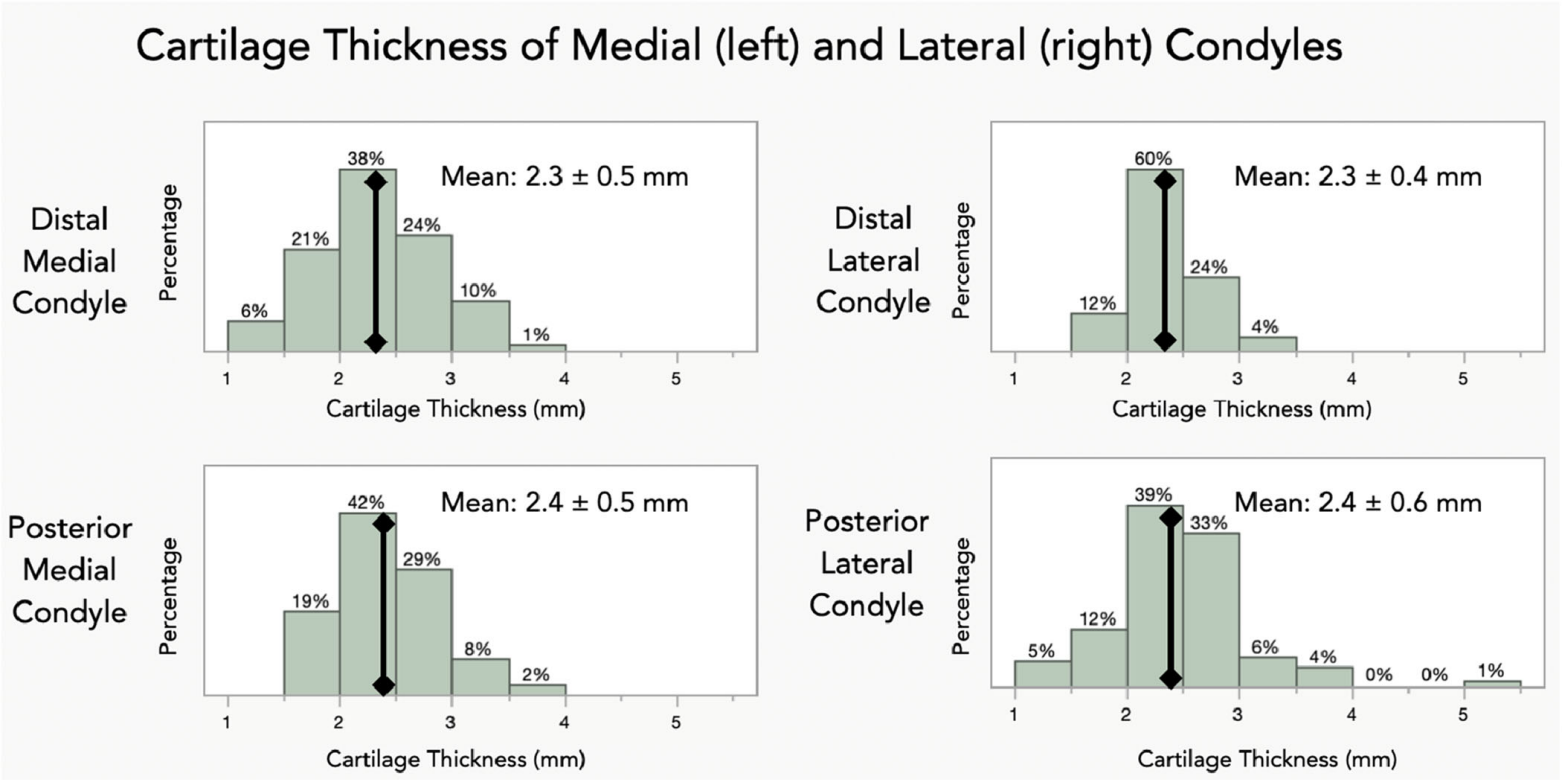
Histograms showing the distribution of cartilage thickness for the distal medial, posterior medial, distal lateral and posterior lateral apices of femoral resections.

For MR images from 100 patients in the OAI database and free from OA, the overall mean apex cartilage thickness was closer to 2.5 mm than 2 mm and compared closely for all four resections (Table 1, Figure 3). Overall means of distal resections were 2.3 mm whereas overall means of posterior resections were 2.4 mm; hence differences were limited to 0.1 mm.

**Table 1:** Summary of results for overall mean apex cartilage thickness measurements from MR images and from calibrated photographs.

	MR images	Calibrated photographs
Distal lateral cartilage thickness	2.3 mm ± 0.4 mm LCL/UCL: 2.2 mm/2.4 mm	2.7 mm ± 0.6 mm (*n* = 155) LCL/UCL: 2.4 mm/2.8 mm
Distal medial cartilage thickness	2.3 mm ± 0.5 mm LCL/UCL: 2.2 mm/2.4 mm	2.6 mm ± 0.7 mm (*n* = 47) LCL/UCL: 2.6 mm/2.7 mm
Posterior lateral cartilage thickness	2.4 mm ± 0.6 mm LCL/UCL: 2.3 mm/2.6 mm	2.5 mm ± 0.5 mm (*n* = 157) LCL/UCL: 2.4 mm/2.5 mm
Posterior medial cartilage thickness	2.4 mm ± 0.5 mm LCL/UCL: 2.3 mm/2.5 mm	2.7 mm ± 0.6 mm (*n* = 97) LCL/UCL: 2.6 mm/2.8 mm

*Note*: 95% confidence limits are also given where LCL and UCL denote lower and upper confidence limits, respectively.

In assessing differences in the overall mean apex cartilage thickness between MR images and calibrated photographs, values were bounded by 0.4 mm for all resections (Table 1). Nevertheless, the t‐tests revealed that differences were statistically significant (*p* ≤ 0.0035) except for the posterior lateral cartilage thickness (*p* = 0.1498). As a result, 95% confidence intervals overlapped only for this overall mean apex thickness (Table 1).

Results for the ICCs indicated excellent agreement with ICCs for intraobserver being >0.95 for all resections and ICCs for interobserver being >0.91 for all resections. Repeatability was markedly better for the posterior resections, showing values of 0.06 mm and 0.04 mm for the posterior medial and posterior lateral resections, respectively, compared to 0.14 mm and 0.10 mm for distal medial and distal lateral resections, respectively. Interobserver variability ranged from 0.03 mm for the posterior medial resection to 0.18 mm for the distal medial resection.

## DISCUSSION

The most important finding was that overall mean apex cartilage thickness based on MR images and based on calibrated photographs was closer to 2.5 mm than 2 mm for all four resections. A second important finding was that overall mean apex thickness varied by only 0.1 mm for the four resections for both measurement methods. Two other important findings were that overall mean apex thickness was significantly greater from calibrated photographs taken of actual resections than measured from MR images and the new MR image‐based method yielded overall mean apex cartilage thickness, which was markedly greater than that reported by other MR image‐based methods.

Our finding that overall mean apex cartilage thickness was closer to 2.5 mm than 2 mm has possible clinical implications. When conceived, the goal of KA was to restore native limb and knee alignments [9]. A requirement for meeting this goal is that the distal femoral and posterior femoral joint lines be restored to native. In an OA knee however, meeting this requirement requires that the cartilage thickness on the worn femoral articular surface(s) be determined and the resection thickness adjusted accordingly. Since this thickness is unknown however, a fixed adjustment of 2 mm has been used but results herein indicate that no confidence intervals included this value (Table 1). Accordingly, a refinement of interest to an already highly successful surgical procedure is to increase the fixed adjustment value.

Since overall mean apex thickness differed significantly between MR images and calibrated photographs, the question arises as to which mean should be used to reflect an increase. To answer this question, it should first be recognised that overall mean apex thickness varied by only 0.1 mm for both measurement modalities. Accordingly, an adjustment for apex cartilage thickness can be the same for all resections. This simplifies the surgical procedure using manual instruments since the same adjustment can be used for both distal and posterior femoral referencing guides.

Considering the difference in means between the two measurement methods, it is worth referring to previous studies to help answer this question. Two recent studies measured cartilage thickness at the apices using a scale [3] and a depth gauge‐femoral referencing guide system [14]. Based on measurements from 189 knees, the study using a scale reported a mean apex thickness of 2.7 mm with a range of 1.5–5 mm, whereas the study using a depth gauge‐femoral referencing guide system based on 100 resections reported a mean apex thickness 2.5 mm with a range of 1.0–5 mm. Hence, given the totality of evidence, increasing the fixed adjustment to 2.5 mm is a refinement of interest. To implement this refinement, as explained in the Methods section once cartilage is removed to the subchondral bone on the worn articular surface, a shim is added to the femoral referencing guide to adjust for cartilage thickness. Accordingly the thickness of the shim simply would be increased.

To appreciate the effects of this refinement, consider a normal distribution for apex thickness with a mean of 2.5 mm and a standard deviation of 0.6 mm (Table 1). From this distribution, the proportion of patients with ≥1 mm side‐to‐side difference reduces from 20% with a fixed 2 mm adjustment to 5% with a fixed 2.5 mm adjusment. Since 5% is the proportion of patients who naturally have this side‐to‐side difference based on the results from the MR images herein, this refinement increases the proportion of patients closer to the ideal KA alignment.

That the overall mean apex thickness from calibrated photographs was generally greater than the overall mean apex thickness based on MR images is an interesting finding that merits discussion. Since MR image‐based cartilage thickness measurements herein were made on healthy knees (Kellgren‐Lawrence Grade 0), whereas thickness measurements from calibrated photographs were made on actual femoral resections where patients had end‐stage OA (Kellgren–Lawrence Grades 3 and 4), the question arises as to whether OA might have affected cartilage thickness in the unworn articular surfaces.

The literature is conflicting in this regard. One longitudinal study analysed MR images of 75 OA knees with a 24‐month follow‐up. Results revealed a substantial portion having cartilage thinning in the central region of the medial (cMF) and lateral (cLF) femur (29%, 13% cMF and cLF, respectively) or thickening (7%, 17% cMF and cLF, respectively) [2]. In contrast, another study evaluated cartilage thickness of the posterior medial and lateral femoral condyles in 227 OA knees with Kellgren‐Lawrence Grade II and greater and 308 non‐OA knees and showed a statistically significant increase in cartilage thickness of the posterior medial femoral condyle for OA knees (2.4 mm vs 2.1 mm) but not the posterior lateral femoral condyle [17]. The authors noted that the lack of a statistically significant increase in cartilage thickness for OA knees in the posterior lateral condyle could be explained by the higher frequency of cartilage lesions on that condyle compared to the posterior medial condyle. In any case, based on these two studies, no firm conclusion is possible regarding the effects of OA on cartilage thickness of the unworn articular surfaces.

Although increasing the fixed adjustment for worn cartilage to 2.5 mm in KA TKA is a refinement of interest, increased adjustment might not be reflected in patient‐reported outcome measures (PROMs). This is because PROMs following KA are relatively insensitive to changes in knee function, which restore function closer to native [4, 5, 6, 10]. Furthermore, using KA‐optimized components, a recent study showed that a fixed 2 mm adjustment did not adversely affect PROMs when apex cartilage on unworn surfaces was ≥3 mm [16]. Another study determined position and orientation deviations of femoral joint lines following KA TKA from virtually planned joint lines and whether these alignment deviations affected PROMs [15]. Alignment deviations were bounded at 2° for most knees. Median FJS and OKS were relatively high, and alignment deviations did not correlate with lower PROMs.

To determine a fixed adjustment for cartilage thickness in KA TKA, an earlier MR image‐based study sought to measure cartilage thickness at the apices of femoral resections and reported markedly lower thicknesses on unworn surfaces [13]. Mean values were 1.8 mm for distal lateral, 1.9 mm for posterior lateral, 1.5 mm for distal medial and 1.9 mm for posterior medial. Since the mean cartilage thickness, rounded to the nearest 0.5 mm, was 2.0 mm on three of the four resections and since means largely agreed with other MR image‐based studies [7, 12], the study concluded that an adjustment of 2.0 mm was reasonable, and this has been the fixed adjustment since that study was published. Although kinematic planes were established similar to those herein and used to identify apices of resections, limitations were that cartilage thickness was measured at a single point on a condyle, and that point was the medial‐lateral ‘centre’ of the condyle. Since no method was described to identify the centre, the point may not have coincided with the apex of a resection. Recognising these limitations, the method used in the current study was developed to overcome these.

Since MR images from the OAI database were used, one limitation of our study is that limb and knee alignments are unknown. Since varus limbs are three times more common than valgus limbs, the sample of 100 patients included approximately 25 valgus limbs. Accordingly, our results can be considered to apply generally to both deformities.

## CONCLUSIONS

Using a new method based on MR images to determine cartilage thickness in knees free from OA, the overall mean apex cartilage thickness was closer to 2.5 mm than 2 mm and was the same for all four resections. Based on calibrated photographs, the overall mean apex thickness was greater than 2.5 mm and was comparable for all four resections. Although a fixed adjustment for worn cartilage on the femur in KA TKA of 2.5 mm for all four resections is a refinement of interest to bring more patients closer to the ideal alignment, this refinement is unlikely to affect patient‐reported outcome measures.

## AUTHOR CONTRIBUTIONS

Saahil Sandhar performed the measurements of cartilage thickness. Alexander J. Nedopil developed the methods and processed data. M. L. Hull conceived the study, developed the methods and wrote the manuscript.

## CONFLICT OF INTEREST STATEMENT

Alexander J. Nedopil receives royalties from and is a paid speaker by Medacta. He is also on the editorial board of *Knee Surgery, Sports Traumatology, and Arthroscopy*. M. L. Hull receives research funding from and is a paid speaker by Medacta. He is also on the editorial boards of *Knee Surgery, Sports Traumatology, Arthroscopy* and the *Journal of Biomechanics*. He is a section editor for *Bioengineering*.

## ETHICS STATEMENT

None declared.

## Data Availability

Data available on request from the authors.
